# Parenting Stress, Parental Self-Efficacy, and Coparenting: Prospective Associations with Child Metabolic Health

**DOI:** 10.1007/s10826-026-03356-4

**Published:** 2026-09-08

**Authors:** Alp Aytuglu, Jennifer E. Graham-Engeland, Mark E. Feinberg, Damon E. Jones, Caesar Liu, Hannah M. C. Schreier

**Affiliations:** 1 Department of Biobehavioral Health, The Pennsylvania State University, University Park, PA, USA; 2 Center for Healthy Aging, The Pennsylvania State University, University Park, PA, USA; 3 Edna Bennett Pierce Prevention Research Center, The Pennsylvania State University, University Park, PA, USA

**Keywords:** Parenting Stress, Parental Self-efficacy, Coparenting, Child Metabolic Health, Child Adiposity

## Abstract

Rising rates of childhood obesity are a serious public health concern, and the family environment plays a central role in shaping early health trajectories. Less is known, however, about how early parenting adjustment and coparenting—both of which help lay the foundation for a nurturing environment—relate to children’s metabolic health. We examined the associations between first-time fathers’ and mothers’ parenting stress and parental self-efficacy during infancy and children’s metabolic health in middle childhood, and tested whether coparenting quality in toddlerhood mediated these associations. Participants were 314 families from the [masked], a randomized controlled trial on the transition to parenthood (identifier: [masked]). Parenting stress and parental self-efficacy were assessed at 10 months, coparenting quality at 24 months, and child metabolic outcomes (glycated hemoglobin [HbA1c], body mass index [BMI], and waist circumference) at ~ 7 years. Greater maternal stress and lower efficacy were associated with lower mother-reported coparenting, which in turn predicted greater child HbA1c and BMI. Although paternal stress and efficacy were associated with father-reported coparenting, no significant pathways to child outcomes emerged for fathers. Findings highlight that maternal perceptions of parenting and coparenting relate to children’s metabolic health and point to the need for continued research on fathers’ roles. Programs and interventions supporting parenting adjustment among first-time parents, especially those that strengthen coparenting, may promote healthier long-term outcomes for children.

## Highlights

First-time fathers’ and mothers’ parenting stress and parental self-efficacy in infancy were associated with coparenting quality in toddlerhood.

Mother-reported coparenting quality in toddlerhood was associated with child HbA1c (glycated blood glucose) and body mass index (BMI) in middle childhood.

Mother-reported coparenting quality in toddlerhood mediated associations between early parenting stress, parental self-efficacy, and child metabolic health in middle childhood.

Pediatric metabolic health is an important focus in child health research, with rising rates of childhood obesity and metabolic dysregulation posing long-term risks for chronic disease (Hu et al., 2025; Ogden et al., 2014; Singer & Lumeng, 2017). Existing research shows that children’s metabolic outcomes are predicted by a range of family-level risk factors, including socioeconomic disadvantage (Miller & Chen, 2010), disruptions in daily family routines (e.g., irregular sleep or mealtimes; Aytuglu et al. 2025a, b; Moens et al. 2007), and poor dietary habits (Walsh et al., 2019). These studies have been instrumental in identifying how structural and behavioral aspects of family life contribute to children’s physical health. Yet, much less attention has been directed toward the role of parental adjustment and interparental relationship quality, particularly during the transition to parenthood, despite their potential relevance for children’s long-term health (Michaelson et al., 2021; Repetti et al., 2002).

The ways in which parents adapt to the demands of their new caregiving role—including how they manage parenting stress and develop a sense of parental self-efficacy (Crnic & Ross, 2017)—may set the stage for early family functioning patterns that influence both the quality of the coparenting relationship (Solmeyer & Feinberg, 2011) and children’s emerging health trajectories (e.g., Kitzman-Ulrich et al., 2010; Mazzeschi et al., 2013). Despite growing recognition of these family processes in adolescence and young adulthood, limited research has examined how early indicators of parental adjustment relate to young children’s metabolic health or whether these links operate through interparental dynamics. To address this gap, the present study investigates whether first-time fathers’ and mothers’ parenting stress and parental self-efficacy during infancy predict two metabolic health outcomes (adiposity and blood glucose regulation) in middle childhood. In addition, we test whether perceived coparenting quality at 24 months mediates the associations between these early parenting adjustment factors and children’s later metabolic outcomes.

## Parenting Stress, Parental Self-Efficacy, and Child Health

Becoming a parent for the first time can be both joyful and challenging. From a family systems perspective, the transition to parenthood is not experienced in isolation but unfolds within an interconnected set of relationships in which changes in one part of the system (e.g., parenting and coparenting) can influence others (Cox & Paley, 2003; Minuchin, 1985). Difficulties adjusting to the parenting role—such as feeling overwhelmed, stressed, or uncertain about parenting skills—may hinder parents’ ability to create stable and responsive caregiving environments. These challenges may negatively affect children’s well-being, including disruptions in physiological regulation (e.g., sleep patterns) and health-related behaviors (e.g., eating routines, physical activity) (Aytuglu et al. 2025a, b; Ekim 2016; Haapala et al. 2022). As outlined in Belsky’s (1984) Determinants of Parenting model, parents’ adjustment is shaped by their psychological resources (coping with stress), infant characteristics (temperament), and contextual factors (coparenting). For example, caring for a temperamentally difficult infant or navigating a strained interparental relationship can further compromise caregivers’ ability to foster nurturing environments (e.g., Huang et al., 2026). These stress-laden contexts in early childhood may initiate a cascade of interrelated processes in a family system (e.g., parenting stress spilling over into coparenting dynamics and caregiving processes) (Cox & Paley, 2003; Liu et al., 2025) that undermine children’s developing self-regulation and increase their vulnerability to long-term physical health problems.

Parenting stress, one core dimension of parental adjustment, has been widely studied in relation to socioemotional development, and a growing body of research now addresses its implications for physical health as well, mostly in older children (Parkes et al., 2019; Parks et al., 2012). Parenting stress arises when caregiving demands exceed a parent’s coping capacity and is often exacerbated by psychological distress, difficult child behavior, or conflict within the interparental relationship (Crnic & Ross, 2017; Deater-Deckard, 1998). Although relatively few studies have examined how parenting stress affects metabolic outcomes in early and middle childhood, emerging evidence—primarily from late childhood and adolescence samples (*M*_age_ = ~ 11 years of age)—suggests that early family stressors may increase children’s risk for obesity and type 2 diabetes (Kim et al., 2019; Mayer-Davis et al., 2017). For example, Parks and colleagues (2012) found that higher levels of parent-reported stress were associated with increased child BMI as well as more frequent fast-food consumption in a sample of children aged 3–17 years—both of which elevate the risk of chronic conditions, such as type 2 diabetes, hypertension, and elevated cholesterol (Juonala et al., 2011). Further, stress in the caregiving environment can also shape children’s physiological regulatory systems, including those involved in cortisol production and glucose metabolism (Carlsson et al., 2014; Gunnar & Donzella, 2002). Although some degree of parenting stress is normative and expected during the transition to a new baby (Epifanio et al., 2015; Favez et al., 2024) and may support the development of regulatory capacities when it is manageable, more chronic or elevated parenting stress may place sustained demands on children’s physiological systems. Prolonged activation of children’s stress-response systems may be associated with alterations in cortisol regulation and glucose metabolism, with downstream implications for metabolic functioning. Over time, these alterations may contribute to children’s risk for metabolic dysregulation (Jimenez et al., 2021; Pervanidou & Chrousos, 2012) and set the stage for patterns of pediatric obesity and insulin resistance that can persist into later developmental stages.

Conversely, parental self-efficacy—the confidence in one’s ability to navigate parenting challenges and support child development—has been linked to more adaptive physiological and behavioral outcomes in children (Coleman & Karraker, 2000). Parents with higher parental self-efficacy are more likely to engage in consistent and structured caregiving practices, reinforcing a stable home environment that supports child well-being (Favez et al., 2024; Merrifield & Gamble, 2013). Although limited research has directly examined the impact of parental self-efficacy on child metabolic health, greater parental self-efficacy has been linked with health-promoting parenting behaviors, including effective communication about nutrition, physical activity, and emotion regulation (Albanese et al., 2019; Ekim, 2016; Haapala et al., 2022). These behaviors may contribute to healthier weight regulation (Heerman et al., 2017; Walsh et al., 2019) and promote more adaptive stress and immune functioning in children (i.e., Miller et al., 2014, 2017). Despite the scarcity of longitudinal research, one intervention study with African American families living in rural poverty (Miller et al., 2014) examined the long-term effects of a parenting program designed to enhance nurturing involvement, behavioral monitoring, and communication around family rules and substance use. Eight years after the intervention, youth in the intervention group showed significantly lower levels of inflammatory markers compared to controls (Miller et al., 2014); the program led to significant improvements in parenting skills, and these improvements were inversely associated with adolescent inflammation. Although this study was conducted in a population (approximately 11-year-old Black American adolescents from low-income households living in the rural South) that differs from the present sample, it provides evidence for broader mechanisms through which parenting processes, such as nurturing caregiving environments, may be linked to biological health.

Early childhood, particularly the first few years of development when children are highly influenced by the caregiving environment, represents a sensitive period for the development of physiological regulatory systems that shape biological health across the lifespan (Gunnar & Donzella, 2002; see also Zuckerman, 2012). Children’s stress regulation, sleep patterns, and health-related routines become established within caregiving environments during this time, forming a foundation for subsequent physiological and behavioral functioning (e.g., Pervanidou & Chrousos, 2012; Ramos et al., 2025). Disruptions in these early family processes may contribute to emerging patterns of physiological dysregulation, including altered stress reactivity and health behaviors, which have been linked to later metabolic risk (Jimenez et al., 2021; Singer & Lumeng, 2017). Accordingly, examining family dynamics during these early developmental periods may provide insight into the origins of metabolic health trajectories.

In summary, there is growing interest in how parenting stress and parental efficacy relate to child metabolic health, although most of the research in this area has focused on adolescent populations. However, the mechanisms linking stress and efficacy to child metabolic health remain unclear. Specifically, it is not well understood how parenting stress and parental efficacy interact with broader family processes, such as interparental relationship quality, to predict a child’s health. Given that interparental relationship quality—particularly coparenting—is central to family systems and plays a defining role in parenting behaviors and child well-being (Cox & Paley, 2003; Feinberg, 2003; Minuchin, 1985), it may serve as a key pathway through which parenting experiences contribute to child health outcomes.

### Mediating Role of Coparenting in Linking Parenting Stress, Parental Self-Efficacy and Child Health

In addition to family systems theory, research has increasingly adopted biopsychosocial models of child and parent health to better understand the complex interplay between psychological (e.g., stress) and social factors (e.g., coparenting) in influencing child physiological health outcomes (Engel, 1977). Within this biopsychosocial framework, the family environment serves as a critical context for examining how these factors interact to shape child well-being and biological health. One key social determinant in this process is interparental relationship quality—particularly coparenting, which refers to how two or more caregivers coordinate, support, and communicate in managing parenting responsibilities and making decisions (Feinberg, 2003). As parents establish daily caregiving routines and coordinate responses to children’s emerging autonomy and regulatory needs during toddlerhood, coparenting interactions function as a key executive subsystem that channels the influence of parenting on child outcomes (Cabrera et al., 2014). As such, coparenting during toddlerhood may serve as a pathway linking earlier parental adjustment in infancy (e.g., parental stress and efficacy) to children’s later physiological health by shaping the quality and consistency of triadic (father–mother–child) family interactions that support healthy development.

Empirical work directly linking coparenting to children’s physical health remains scarce. Although studies of interparental relationships and couple functioning are also limited, this literature provides a foundation for understanding children’s physical health in the coparenting context. Research to date has documented that conflict between parents is associated with increased cardiovascular reactivity to stressors (e.g., elevated heart rate and blood pressure in children during or following exposure to interparental conflict) and heightened skin conductance in children, as well as greater parent-reported child health problems (Ballard et al., 1993; El-Sheikh et al., 2001; Gottman & Katz, 1989). These associations appear especially pronounced when children perceive the conflict as threatening or unresolved or are higher in empathy (El-Sheikh & Harger, 2001; Schreier et al., 2024). In contrast, constructive interparental conflict has been linked to fewer child health complaints, with evidence suggesting that coparenting may mediate these associations, in samples of partnered, co-residing families (Zemp et al., 2020). This body of research primarily emphasizes broader couple dynamics, typically within partnered, co-residing families, although coparenting processes may also occur across diverse family structures. However, the impact of interparental conflict on children’s psychosocial development is accentuated when conflict is related to childrearing or takes place in front of the child. Such childrearing conflicts and exposing the child to interparental conflict are central aspects of the coparenting relationship (Grych & Fincham, 1990). Thus, it is plausible that coparenting may be a key factor shaping child metabolic health.

### Examining Parenting Stress and Parental Self-Efficacy in Fathers and Mothers

In their conceptual model of parenting stress and parental self-efficacy, Crnic and Ross (2017) proposed that both stress and efficacy are linked through a transactional process and can influence—and be influenced by—relational dynamics (e.g., coparenting) and child factors. This model emphasizes the importance of incorporating both fathers’ and mothers’ experiences of parenting to develop a more comprehensive understanding of family functioning and child outcomes. Although both parents undergo stress and develop a sense of efficacy during the transition to parenthood, these experiences—and their implications for family dynamics—may differ (Cabrera et al., 2018; Palkovitz et al., 2014; Solmeyer & Feinberg, 2011). For example, parenting stress in fathers has been linked to interparental difficulties (e.g., perceived undermining behaviors, maternal gatekeeping, lack of coordination on daily caregiving tasks; Elliston et al., 2008; Schoppe-Sullivan et al., 2015), whereas in mothers, who often take on a larger share of caregiving roles in early childhood, it also affects daily caregiving responsibilities in addition to interparental dynamics (Dora d’Orsi et al., 2023). Likewise, although greater parental efficacy is generally beneficial, its expression can vary depending on family roles and dynamics. Higher efficacy may be linked to greater initiative in caregiving and expectations of partner support, but it can also heighten sensitivity to exclusion or coparenting conflict (Donithen & Schoppe-Sullivan, 2022; Jones & Prinz, 2005; Palkovitz et al., 2014). Better recognizing these differences between fathers and mothers can clarify how parental interactions are related to coparenting and, ultimately, child metabolic health over time.

### The Present Study

The present study examined the longitudinal associations between early parental adjustment dimensions among first-time parents, coparenting quality, and child metabolic health outcomes. Data were drawn from three developmental periods—infancy (10 months), toddlerhood (24 months), and early school age (7 years). These developmental periods capture key transitions in early development and parenting, including individual-level parental adjustment (parenting stress and parental efficacy) during infancy, when first-time parents are still adapting to caregiving demands; interparental coordination in toddlerhood, which becomes particularly salient as children’s behavior becomes more complex and requires greater joint management; and a period in early school age when metabolic and behavioral patterns become more stable and measurable. Specifically, we investigated whether parenting stress and parental efficacy (reported by fathers and mothers when their child was 10 months old) predicted father- and mother-reported coparenting quality when children were 24 months of age, and whether, in turn, coparenting quality was associated with child glycated hemoglobin (HbA1c) and adiposity (BMI and waist circumference) at approximately seven years of age. We hypothesized that lower parenting stress and greater parental efficacy would be associated with better coparenting quality, which would subsequently predict lower child adiposity and HbA1c levels.

## Method

### Participants

Data for this study were derived from [masked] (*N* = 399 families), a longitudinal randomized, controlled trial focused on coparenting quality and family relationships (see, masked). Participants included 314 families (fathers, mothers, and children; 49.4% girls) from three Mid-Atlantic states (Delaware, Maryland, and Pennsylvania) and one Southern state (Texas). Families were eligible to participate if both parents were at least 18 years old, expecting their first child together, and living together at the time of enrollment. This focus on first-time parents enabled us to capture the emergence of coparenting dynamics and parents’ adjustment to new caregiving roles during the transition to parenthood. Following the initial assessment during pregnancy (wave 1, *n* = 399; mothers averaged 22.8-week gestation, *SD* = 5.5), families were visited in their homes at three follow-up points: approximately 10 months postpartum (wave 2; *n* = 314), 24 months postpartum (wave 3; *n* = 296), and seven years postpartum (wave 5; M_age_ = 7.43 years, *SD* = 0.62; *n* = 164). Participant retention was 78.7% from baseline to wave 2, 94.3% from wave 2 to wave 3, and 55.4% from wave 3 to wave 5, resulting in an overall retention rate of 41.1% from baseline to wave 5. At baseline assessment, fathers had a mean age of 31.09 years (*SD* = 5.36), and mothers had a mean age of 29.12 years (*SD* = 4.40). The majority of parents identified as non-Hispanic White (81%), followed by Hispanic (7%), Black American (6%), and multiracial (2%). Most parents were highly educated, with the majority reporting a bachelor’s degree or higher (73% of mothers and 60% of fathers). Relatively few reported only a high school diploma (5.5% of mothers and 13.6% of fathers) or less than a high school education (1% of mothers and 3% of fathers). Given high stability in family income across waves 1–3 (Spearman’s ρ = 0.77–0.88, all *p*s < 0.001) and substantial attrition at wave 5, we used income at wave 2 to align with the main study variables. Annual family income at wave 2 (10 months) ranged from less than $5,000 to over $150,000, with an average family income between $80,000 and $85,000. At wave 5, the majority of birth parents (87.4%) reported that they were married and living at home with the child, whereas 10% reported that the birth mother lived with the child and the birth father saw the child at least once per month.

### Procedure

This study was approved by the Institutional Review Board of [masked]. At each wave, parents provided informed consent and children provided assent, and families were compensated for their time and effort. The present study focuses on waves 2, 3, and 5, as these waves included the primary constructs of interest. (Baseline [wave 1, a prenatal assessment] and wave 4 [involving only a subset of participants and focused on interparental violence] were not included in the present analyses because they did not assess constructs central to the current study). At wave 2 (10 months), fathers and mothers completed a series of survey questions assessing sociodemographics, parenting stress, and parental self-efficacy. At wave 3 (24 months), they completed survey questions assessing coparenting relationships. At wave 5 (~ 7 years), children’s height and weight were measured to enable calculation of BMI, and waist circumference was measured as an assessment of central adiposity; in addition, children’s capillary whole blood was collected via finger stick and later analyzed for HbA1c.

Prior to blood sample collection in wave 5, all procedures (including blood collection and anthropometric measurements) were explained to children in age-appropriate language to ensure understanding and minimize discomfort. At least one parent remained present with the child throughout all procedures. Capillary whole blood was collected via finger-stick by trained research staff using a single-use micro-lancet after cleaning the puncture site with an alcohol swab. Blood was collected onto filter paper cards (dried blood spots) following standardized procedures designed to minimize discomfort. Samples were air-dried, stored with desiccant, and shipped to a laboratory for HbA1c analysis.

### Measures

#### Parenting and Coparenting Factors

##### Parenting Stress

At wave 2 (10 months), parental stress was assessed using the 27-item Parental stress Index (PSI; Abidin, 1995), completed separately by fathers and mothers. The PSI consists of three subscales: Parental Distress (e.g., *“I feel trapped by my responsibilities as a parent.”*), Parent-Child Dysfunctional Interaction (e.g., *“When playing*,* my child doesn’t often giggle or laugh.”*), and Difficult Child (e.g., *“My child seems to cry or fuss more often than most children.”*). All items were rated on a 5-point Likert scale ranging from 1 (Strongly Disagree) to 5 (Strongly Agree), with higher scores indicating greater parenting stress. Cronbach’s alpha (α) for the total parenting stress score, calculated as the aggregate of all subscale items, indicated strong reliability, with values of α = 0.90 for fathers and α = 0.91 for mothers.

##### Parental Efficacy 

At wave 2 (10 months), parental efficacy was assessed using eight items from the Parenting Sense of Competence Scale (Johnston & Mash, 1989). This measure evaluates how new parents think about their role and confidence in parenting. Sample items include *“I feel confident in my role as a parent”* and *“In most circumstances*,* even when I am tired*,* I am able to cope well with meeting my baby’s needs”.* Parents responded on a 7-point Likert scale ranging from 1 (Not at all like me) to 7 (Completely like me). Item scores were summed to create a total score, with higher scores reflecting greater parental efficacy. Cronbach’s alpha (α) values were good, at α = 0.85 for fathers and α = 0.95 for mothers.

##### Coparenting Relationship 

At wave 3 (24 months), coparenting quality was assessed using the Coparenting Relationship Scale (CRS; Feinberg et al., 2012). This 33-item measure yields an overall coparenting score and includes six subscales: coparenting agreement (e.g., *“My partner and I have the same goals for our child.”*), coparenting closeness (e.g., *“My relationship with my partner is stronger now than before we had a child.”*), coparenting support (e.g., *“My partner appreciates how hard I work at being a good parent.”*), coparenting undermining (e.g., *“My partner does not trust my abilities as a parent.”*), endorsement of partner’s parenting (e.g., *“My partner pays a great deal of attention to our child.”*), and child exposure to conflict (e.g., *“Yell at each other within earshot of the child?”*). Items were rated on a 7-point Likert scale from 0 (Not true of us) to 6 (Very true of us) for the first five subscales, and child exposure to conflict items were rated from 0 (Never) to 6 (Very often, several times a day). Higher scores indicate more positive coparenting. Cronbach’s alpha (α) values for the overall coparenting score were good, at α = 0.87 for fathers and α = 0.88 for mothers. 

#### Child Adiposity

##### BMI

At wave 5 (~ 7 years), children’s height and weight were measured using a portable stadiometer and a digital scale, respectively. BMI (kg/m²) was calculated as weight divided by height squared and then converted to an age- and sex-specific percentile based on standardized growth charts (Kuczmarski et al., 2002). Higher BMI percentiles indicate greater adiposity relative to same-age, same-sex peers. In the current sample, the mean BMI percentile was 56.0 (*SD* = 28.5), indicating considerable variability in BMI distribution.

##### Waist Circumference

 At wave 5, children’s waist circumference was measured at the midpoint between the hip bone and the lowest rib using a cloth tape. Waist circumference is a commonly used indicator of central adiposity and has been associated with increased cardiometabolic risk in children and adults (Shen et al., 2006).

##### HbA1c

HbA1c is a reliable indicator of long-term glycemic control, with higher levels reflecting poorer regulation (Pollock et al., 2016). At wave 5, blood was collected from children’s nondominant middle or ring finger using a single-use micro-lancet (BD Microtainer), with five free-flowing drops placed onto filter paper cards, air-dried for four hours, and shipped to [masked] laboratory at [masked]. The cards were then stored at − 30 °C until being sent in batches to the Department of Laboratory Medicine at the University of Washington for HbA1c analysis. Measurements followed established protocols and were conducted using an ion-exchange high-performance liquid chromatography system (Bio-Rad Variant II, Bio-Rad Laboratories Inc., Clinical Systems Division, Hercules, CA), a widely recognized method known for its high sensitivity and accuracy (Maleska et al., 2017; Pollock et al., 2016).

### Analytical Plan

We first examined descriptive statistics and bivariate correlations among study variables. Next, we used structural equation modeling (SEM) to test the hypothesized direct and indirect associations between parental stress, parental self-efficacy, coparenting quality, and child adiposity (HbA1c, BMI, and waist) in Mplus Version 8.10 (Muthén & Muthén, 2012–2023). Mplus utilizes full information maximum likelihood (FIML) estimation to account for missing data, which enables full sample estimation by including all available non-missing data under the assumption that data are missing at random, conditional on the observed variables (Enders & Bandalos, 2001; MacKinnon et al., 2004). Bias-corrected bootstrapping procedures were used to estimate indirect effects, as this approach is generally preferred given that it provides power benefits relative to other tests of mediation and does not require a significant direct association between the independent variable and outcome to interpret indirect effects (Enders & Bandalos, 2001; Fritz & MacKinnon, 2007; MacKinnon et al., 2004). Model fit was evaluated based on established criteria (Hu & Bentler, 1999), including χ², the comparative fit index (CFI ≥ 0.90), root mean square error of approximation (RMSEA ≤ 0.08), and standardized root mean square residual (SRMR ≤ 0.08). Indirect effects were considered statistically significant if 95% confidence intervals (CIs) did not contain zero. All models adjusted for child gender, parental educational attainment, family income, and intervention group status (1 = intervention group, 0 = comparison group) when predicting coparenting quality and child metabolic health variables.

## Results

Descriptive statistics, including means, standard deviations, and zero-order correlations among all continuous study variables, are presented in Table 1. Preliminary analyses indicated that mother-reported coparenting quality was significantly and negatively correlated with child BMI and was marginally negatively associated with child HbA1c. All but one bivariate correlation between father- and mother-reported parenting stress, parental efficacy, and coparenting quality were statistically significant and in the expected directions. Regarding covariates, family income was negatively correlated with both father and mother parenting stress, and positively correlated with father parental efficacy as well as father and mother coparenting quality. Both father and mother education were positively correlated with coparenting quality, and father education was positively correlated with child waist circumference. Independent-samples t tests revealed a significant difference in child waist circumference (*p* < .01) based on child gender, with girls having smaller waist circumference than boys (*M*_girls_ = 57.12 cm; *M*_boys_ = 61.59 cm). Further, independent samples t-tests and chi-square analyses were also conducted to examine differences between families retained at wave 5 and those not retained at wave 5. These analyses indicated no significant differences in demographic characteristics or most study variables (all *p*s > 0.05). The only significant difference observed was that fathers in families retained at wave 5 reported slightly lower parental efficacy at wave 2 compared to those not retained, *t*(303) = 2.13, *p* = .03.

**Table 1:** Correlation Between Parenting Stress, Parental Self-Efficacy, Coparenting, and Child Health Outcomes

Variable	M		SD	1	2	3	4	5	6	7	8	9	10	11	12
**IVs**															
1. Paternal Parenting Stress		1.79	0.48	—											
2. Maternal Parenting Stress		1.80	0.62	0.36**	—										
3. Paternal Self-Efficacy		46.29	6.42	− 0.62**	− 0.26**	—									
4. Maternal Self-Efficacy		48.04	6.10	− 0.27**	− 0.55**	0.20**	—								
**Mediators**															
5. Father Coparenting		4.91	0.66	− 0.36**	− 0.25**	0.25*	0.14**	—							
6. Mother Coparenting		5.04	0.81	− 0.15*	− 0.21**	**0.08**	0.15**	0.62**	—						
**DVs**															
7. Child HbA1c		5.14	0.33	− 0.01	− 0.02	− 0.04	− 0.02	− 0.10	− 0.20^†^	—					
8. Child BMI (percentile)		55.66	28.56	0.04	0.14	0.05	− 0.09	− 0.03	− 0.18*	0.06	—				
9. Child Waist (cm)		59.46	8.72	− 0.06	− 0.00	− 0.02	0.02	− 0.02	0.02	0.05	0.40**	—			
**Covariates**															
10. Family Income		16.32^a^	5.49	− 0.16*	− 0.15*	0.13*	− 0.01	0.13*	0.16**	0.03	− 0.02	0.01	—		
11. Father Education		15.09^b^	1.89	− 0.11	− 0.05	0.00	0.00	0.19**	0.31**	− 0.11	− 0.15	− 0.02	0.39**	—	
12. Mother Education		15.66^b^	1.54	− 0.07	0.00	0.01	0.00	0.15**	0.22**	0.01	0.05	0.13	0.44**	0.47**	—
***N***				306	314	305	313	296	296	104	146	164	309	398	398

^†^*p*<.07, *p< .05, **p< .001. *M* = Mean Scores; *SD* = Standard Deviations; IVs = Independent Variables; DVs = Dependent Variables; a = Income brackets 16 (i.e., 80,000–90,000); b = Parent educational attainment in years

### Hypothesis Testing

#### Parenting Stress, Coparenting, and Child Metabolic Health

**Fig. 1:**
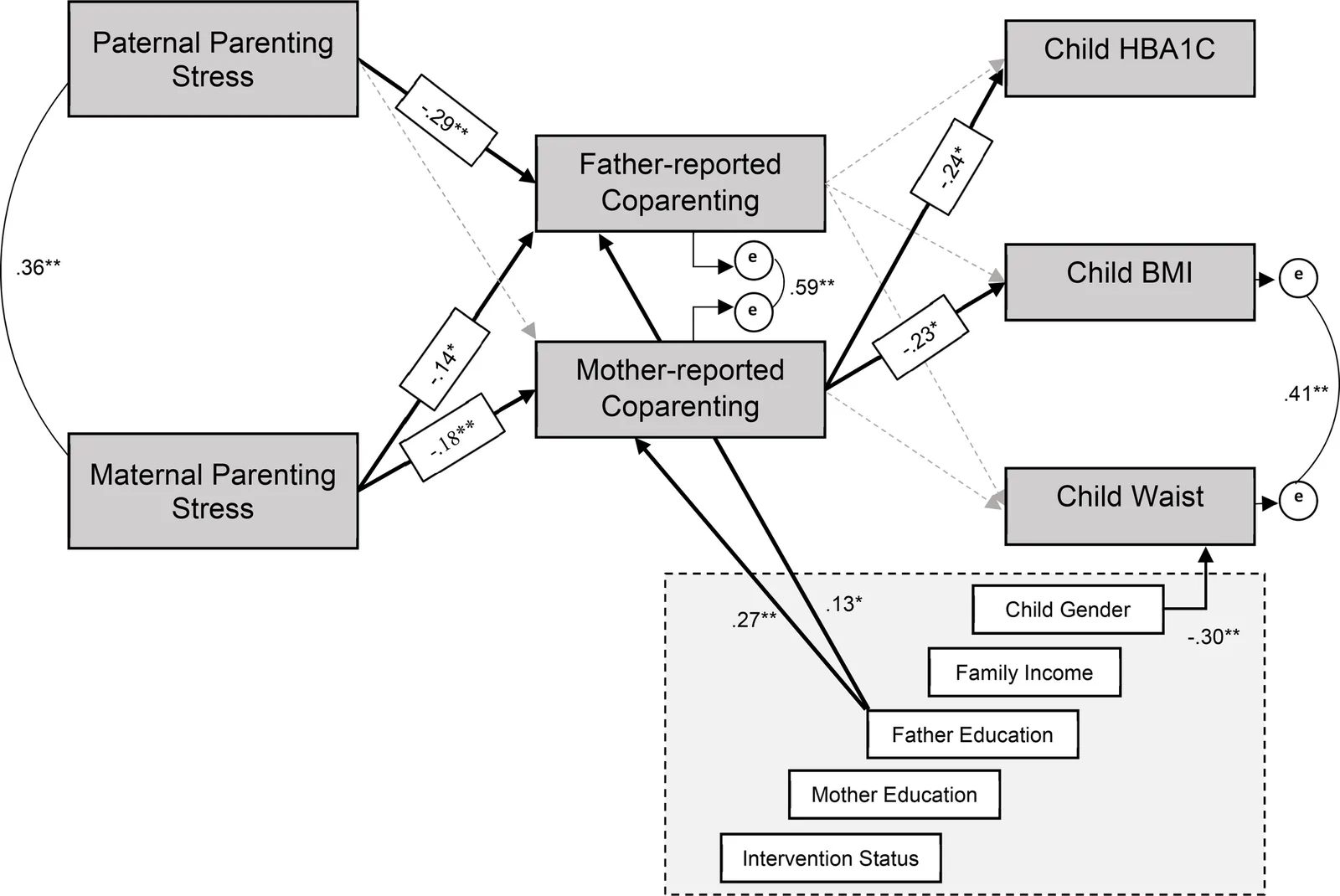
Coparenting as a Mediator Between Parenting Stress and Child Health Outcomes. Note. *p < .05, **p < .01. Solid black lines and dashed gray lines indicate significant and non-significant paths, respectively. Standardized coefficients are presented. The gray area with a dashed border includes covariates. Non-significant paths from parenting stress to health outcomes and from covariates to study variables were omitted for clarity. The specific indirect effects from mothers’ parenting stress to child HbA1c (95% CI [0.007, 0.109]) and BMI (95% CI [0.002, 0.099]) via mother-reported coparenting are significant, as the confidence intervals do not include zero

A structural equation model was estimated in which father- and mother-reported parental stress at 10 months predicted coparenting quality at 24 months, as well as three child metabolic health outcomes (HbA1c, BMI, and waist circumference) at ~ 7 years, with child gender, parental educational attainment, family income, and intervention status included as covariates predicting coparenting and the child outcomes (see Fig. 1). Model fit indices indicated good fit: *χ²*(16) = 24.782, *p* = .07, RMSEA = 0.04 (90% CI [0.00, 0.07]), CFI = 0.96. Table 2 presents the standardized coefficients, standard errors, and associated p-values for all indirect structural path coefficients in this model.

**Table 2:** Standardized Indirect Effect, Standard Errors, and Bootstrapped 95% Confidence Intervals

Indirect Effect	Estimate	SE	[95% CI]
Parenting Stress Model			
Maternal Stress T2 → M-R Coparenting T3 → Child HbA1c	**0.043**	**0.024**	**[0.007**,** 0.109]**
Maternal Stress T2 → M-R Coparenting T3 → Child BMI	**0.042**	**0.025**	**[0.002**,** 0.099]**
Maternal Stress T2 → M-R Coparenting T3 → Child Waist	− 0.006	0.020	[-0.052, 0.029]
Paternal Stress T2 → F-R Coparenting T3 → Child HbA1c	− 0.008	0.043	[-0.100, 0.077]
Paternal Stress T2 → F-R Coparenting T3 → Child BMI	− 0.046	0.041	[-0.142, 0.024]
Paternal Stress T2 → F-R Coparenting T3 → Child Waist	0.032	0.041	[-0.041, 0.123]
Parental Self-Efficacy Model			
Maternal Efficacy T2 → M-R Coparenting T3 → Child HbA1c	**− 0.034**	**0.022**	**[-0.092**,** − 0.001]**
Maternal Efficacy T2 → M-R Coparenting T3 → Child BMI	− 0.030	0.022	[-0.086, 0.002]
Maternal Efficacy T2 → M-R Coparenting T3 → Child Waist	0.002	0.016	[-0.026, 0.039]
Paternal Efficacy T2 → F-R Coparenting T3 → Child HbA1c	0.012	0.035	[-0.050, 0.090]
Paternal Efficacy T2 → F-R Coparenting T3 → Child BMI	0.025	0.032	[-0.030, 0.105]
Paternal Efficacy T2 → F-R Coparenting T3 → Child Waist	− 0.020	0.032	[-0.091, 0.038]
Maternal Stress and Efficacy Model			
Maternal Stress T2 → M-R Coparenting T3 → Child HbA1c	**0.037**	**0.025**	**[0.002**,** 0.104]**
Maternal Stress T2 → M-R Coparenting T3 → Child BMI	0.026	0.020	[-0.002, 0.080]
Maternal Stress T2 → M-R Coparenting T3 → Child Waist	0.006	0.014	[-0.016, 0.038]
Maternal Efficacy T2 → M-R Coparenting T3 → Child HbA1c	− 0.013	0.019	[-0.055, 0.021]
Maternal Efficacy T2 → M-R Coparenting T3 → Child BMI	− 0.009	0.016	[-0.051, 0.014]
Maternal Efficacy T2 → M-R Coparenting T3 → Child Waist	− 0.002	0.008	[-0.030, 0.006]

Standardized coefficients are presented. SE = standard error; CI = confidence interval; F-R = father report; M-R = mother report; T2 = Wave 2 data collection; T3 = Wave 3 data collection

Fathers’ parenting stress was negatively associated with father-reported coparenting quality (*β*father = − 0.29, *p* < .01), mothers’ parenting stress was negatively associated with mother-reported (*β*mother = − 0.18, *p* < .01) and father-reported (*β*mother = − 0.14, *p* < .05) coparenting quality. Furthermore, mother-reported coparenting quality was negatively associated with child health outcomes, specifically HbA1c (*β* = − 0.24, *p* < .05) and BMI (*β* = − 0.23, *p* < .05), but not waist circumference (*β* = 0.03, *p* = .76). There were no significant direct associations between fathers’ and mothers’ parenting stress and child health outcomes. Also, no significant associations were found between father-reported coparenting quality and child health outcomes. Additionally, two significant indirect effects emerged, indicating that maternal parental stress at 10 months predicted higher child HbA1c levels (95% CI [0.007, 0.109]) and BMI (95% CI [0.002, 0.099]) at age ~ 7 through mother-reported coparenting quality (see Fig. 1).

#### Parental Self-Efficacy, Coparenting, and Child Metabolic Health

**Fig. 2:**
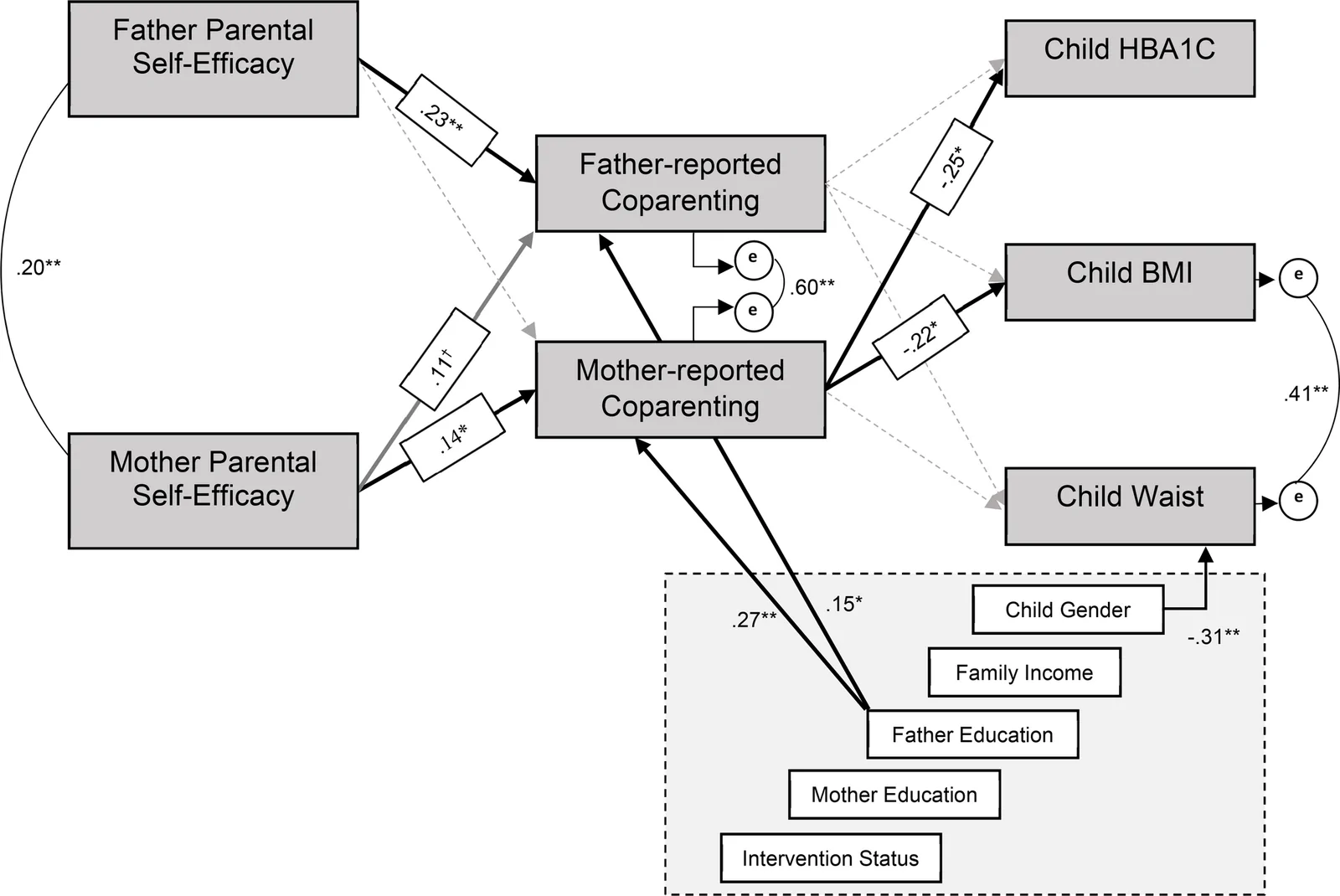
Coparenting as a Mediator Between Parental Self-Efficacy and Child Health Outcomes. Note. † *p* = .07, *p < .05, **p < .001. Solid black lines, solid gray lines, and dashed gray lines indicate significant, marginally significant, and non-significant paths, respectively. Standardized coefficients are presented. The gray area with a dashed border includes covariates. Non-significant paths from paternal self-efficacy to health outcomes and from covariates to study variables were omitted for clarity. The specific indirect effect from mothers’ parental efficacy to child HbA1c (CI [-0.092, − 0.001]) via mother-reported coparenting is significant, as the confidence intervals do not include zero

Next, we estimated a second structural equation model to examine whether parental self-efficacy at 10 months predicted coparenting quality at 24 months and child metabolic health outcomes (HbA1c, BMI, and waist circumference) at ~ 7 years, with child gender, parent educational attainment, family income, and intervention status included as covariates predicting coparenting and the child outcomes (see Fig. 2). Model fit indices indicated good fit: *χ²*(16) = 18.373, *p* = .30, RMSEA = 0.02 (90% CI [0.00, 0.06]), CFI = 0.99. Table 2 presents the standardized coefficients, standard errors, and associated p-values for indirect structural path coefficients in this model.

Fathers’ parental efficacy was positively associated with father-reported coparenting quality (*β*father = 0.23, *p* < .01), whereas mothers’ parental efficacy was significantly and positively associated with mother-reported coparenting quality (*β*mother = 0.14, *p* < .05) and marginally associated with father-reported coparenting quality (*β*mother = 0.11, *p* = .07). In this model, mother-reported coparenting quality was negatively associated with child health outcomes, specifically HbA1c (*β* = − 0.25, *p* < .05) and BMI (*β* = − 0.22, *p* = .05), but not child waist circumference (*β* = 0.01, *p* = .89). These associations were not significant for father-reported variables, and father and mother parental efficacy were also not directly associated with child health outcomes. A significant indirect effect indicated that maternal parental efficacy at 10 months predicted lower child HbA1c levels (95% CI [–0.092, –0.001]) at age 7 through mother-reported coparenting quality (see Fig. 2).

#### Combined Maternal Parental Stress and Self-Efficacy, Coparenting, and Child Metabolic Health

**Fig. 3:**
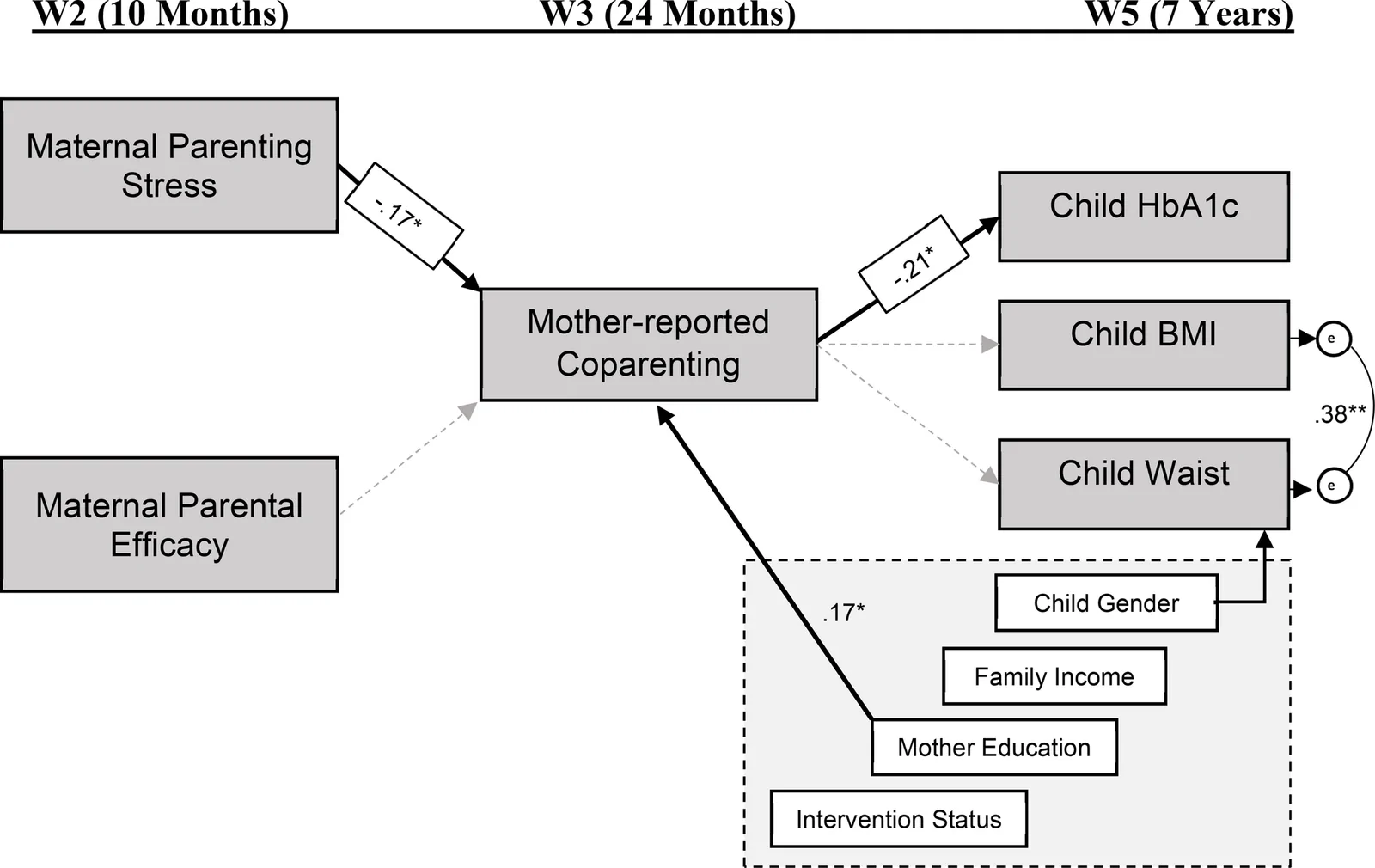
Mother Coparenting as a Mediator Between Maternal Parenting Stress, Maternal Self-Efficacy and Child Health Outcomes. Note. *p < .05. Solid black lines and dashed gray lines indicate significant and non-significant paths, respectively. Standardized coefficients are presented. The gray area with a dashed border includes covariates. Non-significant paths from paternal self-efficacy to health outcomes and from covariates to study variables were omitted for clarity. The specific indirect effect from mothers’ parenting stress to child HbA1c (CI [0.002, 0.104]) via mother-reported coparenting is significant, as the confidence intervals do not include zero

 To build on the findings from the first two models, we estimated a third structural equation model to examine whether maternal parenting stress or self-efficacy was more strongly associated with coparenting and child metabolic health outcomes when entered simultaneously. This follow-up model focused on maternal predictors only, as paternal stress and efficacy were not significantly associated with coparenting or child outcomes in the earlier models (Fig. 3). Model fit indices indicated good fit: *χ²*(3) = 4.031, *p* = .26, RMSEA = 0.04 (90% CI [0.00, 0.11]), CFI = 0.98. In this model, maternal parenting stress at 10 months continued to significantly predict mother-reported coparenting quality at 24 months (*β*mother = − 0.17, *p* < .05). Mother-reported coparenting quality, in turn, predicted child health outcomes at 7 years, including lower HbA1c (*β*mother = − 0.22, *p* < .05) and marginally lower BMI (*β*mother = − 0.15, *p* = .08), but not child waist circumference (*β*mother = − 0.04, *p* = .62). A significant indirect effect was observed, indicating that maternal parental stress at 10 months indirectly predicted greater child HbA1c levels at age *~* 7 through mother-reported coparenting quality (95% CI [0.002, 0.104]).

## Discussion

Guided by family systems theory and the biopsychosocial model of child health (Engel, 1977; Minuchin, 1985), this study examined prospective associations between parental adjustment—parenting stress and parental self-efficacy—and coparenting relationship quality, along with their roles in shaping children’s metabolic health outcomes among first-time parent dyads. Parenting stress and efficacy reported by both mothers and fathers at 10 months were associated with their own perceptions of coparenting quality at 24 months. In turn, mother-reported coparenting quality was associated with lower child BMI and HbA1c levels when children were approximately seven years old. Moreover, in a model including both maternal parenting stress and parental self-efficacy simultaneously, only mothers’ parenting stress significantly and negatively predicted coparenting quality, which in turn was linked to lower child HbA1c levels. Fathers’ parenting stress and parental self-efficacy, as well as father-reported coparenting quality, were not significantly associated with child metabolic health outcomes. This suggests that maternal parenting experiences may play a more salient role in linking early coparenting relationship quality to children’s physiological health.

### Parenting Stress, Parental Self-Efficacy, and Coparenting

Parenting stress in new parents is well-documented to spill over into the broader family system, often disrupting collaborative parenting (Cox & Paley, 2003). This aligns spillover processes within the family system whereby strain in the parenting role extends into the marital or coparenting subsystem (Erel & Burman, 1995; e.g., Liu et al., 2025). In the current study, greater maternal parenting stress at 10 months predicted lower coparenting quality at 24 months, based on both mother- and father-reports. One explanation may be that mothers experiencing greater parenting stress may have reduced emotional capacity to engage in supportive coparenting, increasing the likelihood of coparental conflict (Liu et al., 2025). The family systems framework also highlights crossover processes, wherein one partner’s parenting stress influences the other’s perceptions or behaviors (Cox et al., 2001). For example, a highly stressed mother may exhibit greater irritability or withdrawal toward her partner’s parenting efforts, which may heighten tension between parents and diminish the father’s sense of coparental unity. Findings with maternal parenting stress in the present research are consistent with previous work linking parenting stress to poorer interparental functioning and less harmonious coparenting (Crnic & Ross, 2017).

In contrast to maternal parenting stress, paternal parenting stress in the present research was associated only with fathers’ own perceptions of coparenting, suggesting a more limited and self-referential influence of paternal stress compared to maternal stress. Several factors may account for this pattern. Mothers and fathers may experience their parenting roles differently, especially during infancy, when mothers are more likely to take on the primary caregiving role. As such, maternal stress may more broadly affect both mother- and father-reported coparenting quality. In contrast, fathers may be less likely to verbalize or express stress in ways that are perceived by their partners. This aligns with evidence suggesting that fathers under stress often withdraw or internalize distress rather than directly disrupt the coparenting relationship (Cabrera et al., 2014). Distinct patterns of maternal and paternal coparenting have also been observed in prior work linking coparenting to child outcomes, although the nature of these differences varies across studies. Some findings suggest that fathers’ parenting stress or negative coparenting behaviors may be particularly salient for child adjustment or overall family dynamics. Schoppe-Sullivan and colleagues (2023) found that, although many families showed broadly similar coparenting patterns in their sample, children exhibited the poorest adjustment in profiles characterized by moderate coparenting quality but lower paternal positivity relative to maternal positivity, as reported by each parent. Similarly, Aytuglu and colleagues (2025) found that fathers’ observed competitive-withdrawal coparenting was associated with child health outcomes, whereas comparable maternal pathways were not. Together with the present findings, this pattern may suggest that paternal stress or coparental negativity may be more likely to influence family functioning when it is more clearly expressed in interactions, whereas when such negativity is less apparent, it may remain more confined to fathers’ perceptions.

Reports of parental self-efficacy in the present research mirrored the differential influence of parenting stress by gender on the coparenting relationship. Maternal self-efficacy in infancy positively predicted coparenting quality as perceived by mothers (and, marginally, fathers), whereas paternal self-efficacy was associated only with fathers’ own perceptions of coparenting. Consistent with prior research, mothers’ sense of parenting competence may broadly facilitate cooperative parenting dynamics, especially given their frequent primary caregiving roles in infancy (Feinberg, 2002). Greater maternal self-efficacy likely promotes emotional availability, responsiveness, and supportiveness in interactions with the father, strengthening the coparental relationship as perceived by both partners. In contrast, paternal self-efficacy may predominantly shape fathers’ own perceptions of coparenting, possibly due to their less direct influence on day-to-day coparenting interactions in infancy (Schoppe-Sullivan et al., 2008). Taken together, these findings suggest that maternal parental adjustments—both stress and efficacy—may have broader and more observable implications for parents’ perception of coparenting quality, whereas paternal characteristics may exert subtler, more self-focused effects. These patterns underscore the interdependence of family subsystems and highlight the distinct ways each parent contributes to shaping the coparenting environment.

### Coparenting and Child Health

In line with our hypothesis, the quality of the coparenting alliance in early childhood was linked to two child physical health outcomes after several years. Specifically, more positive mother-reported coparenting quality in toddlerhood was associated with lower HbA1c levels and lower BMI by age seven. This finding aligns with and adds to a growing body of research suggesting that positive family relationships support not only children’s socioemotional development but also their physiological health (Ha & Granger, 2016; Repetti et al., 2002). Developmental and health psychology frameworks (Cox & Paley, 2003) emphasize that the foundations of chronic health conditions—such as obesity and metabolic health problems—are shaped, in part, by early family environmental influences (e.g., Kitzman-Ulrich et al., 2010). A supportive coparenting relationship is a key marker of a nurturing family climate, whereas conflicted or undermining coparenting relationships may constitute a stress-inducing environment for the child (Crnic & Ross, 2017). The present findings suggest that when parents work together harmoniously in raising their children, their children are more likely to exhibit better metabolic health in the early school years. In contrast, lower coparenting quality, as perceived by mothers, was associated with elevated BMI and HbA1c levels, pointing to potential adverse metabolic consequences from a less supportive early family environment.

There are several mechanisms that may help explain how early coparenting processes can translate into biological outcomes that influence a child’s BMI and glucose regulation. One potential pathway is chronic stress and physiological regulation. Greater coparenting quality likely provides the child with a more secure, low-conflict environment. Children in such settings are less likely to experience frequent or intense activation of stress-response systems, which may reduce the adverse effects of chronic stress on metabolic functioning (Gunnar & Quevedo, 2007). By contrast, elevated stress hormones and repeated disruptions to daily routines (e.g., irregular meals or sleep) due to coparenting conflict can contribute to weight gain and impaired glycemic control, as chronic stress has been associated with increased insulin resistance and fat storage (Charmandari et al., 2012). Thus, the socio-emotional security fostered by a positive coparenting relationship may contribute to healthier neuroendocrine and metabolic outcomes for the child.

Another pathway may involve how coparents coordinate around family routines that promote children’s health. When the coparenting relationship is strong, parents are more likely to support each other’s efforts and maintain consistent practices related to diet, physical activity, and daily structure. Although the present study assessed only global perceptions of coparenting alliance rather than specific health-related daily routines, prior research suggests that higher-quality coparenting is linked to greater alignment in these domains. Research indicates that such alignment is linked to more effective implementation of health-promoting routines (e.g., Khandpur et al., 2016; Tan et al., 2021). For example, greater coparenting quality has been associated with greater agreement on child feeding practices and mealtime structure, which in turn is linked to lower child BMI (Ramos et al., 2025). Conversely, in less cooperative coparenting relationships, one parent may undermine established routines or parental disagreements may result in inconsistent limit-setting (e.g., mixed messages about snacks or screen time). Taken together, these represent pathways through which coparenting may be associated with children’s physiological health, although the mediating mechanisms were not directly assessed in the current study. Future research incorporating direct measures of these processes will be important for clarifying these pathways.

Results also showed that mother-reported coparenting quality was associated with children’s BMI but not waist circumference. Although both indices are commonly used to assess adiposity in children and adolescence (Cameron et al., 2009; Mei et al., 2002), relatively little is known about how well they capture differences in younger school-aged children. One possibility is that child BMI, which is adjusted for age and gender, and widely used as a first-line screening tool for general obesity in children (Freedman et al., 2009), may have functioned as a more sensitive indicator in our sample. In contrast, waist circumference is measured in raw centimeters and reflects central adiposity, which may be more difficult to capture reliably at this developmental stage and could become more informative later in childhood or adolescence (Cameron et al., 2009). These interpretations should be viewed as tentative, and future work will be needed to clarify how family processes relate to different adiposity indicators across development.

### Limitations and Future Directions

Several limitations should be considered when interpreting the current findings. First, our assessment of parenting stress was limited to a single time point during infancy. Although chronic or excessive stress is typically considered detrimental, mild or moderate stress may have adaptive or even beneficial effects on parenting and coparenting, potentially by motivating parents to mobilize resources, seek support, or become more attentive and vigilant in their caregiving efforts. Future studies employing intensive longitudinal designs (e.g., multiple daily assessments of parenting stress over several days or weeks) could better capture the nuanced and dynamic role of stress. Second, due to data constraints, we examined parenting stress and efficacy only during infancy as predictors of later coparenting quality and child metabolic health. In addition, although these temporal associations are theoretically plausible—parenting stress tends to be high among first-time parents during infancy (Nomaguchi & Milkie, 2003), whereas coparenting dynamics consolidate as the triadic relationship strengthens in toddlerhood (see Campbell, 2023, for review)—future research is needed to clarify these complex, reciprocal links. Further, although parenting-related processes during pregnancy are often shaped by expectations for the parenting role (e.g., Huang et al., 2026), the present study does not capture prenatal variability in these constructs. Future research may benefit from examining how prenatal factors—such as parents’ expectations about their child, parental well-being, and prenatal parental efficacy—relate to postnatal parenting stress and self-efficacy, and, in turn, to children’s health outcomes. Similarly, metabolic outcomes were available only at 7 years of age, which limits our ability to interpret levels of BMI and HbA1c relative to earlier baseline functioning or to examine developmental changes over time. Overall, longitudinal designs with repeated assessments would enable disentangling reciprocal associations across developmental periods using cross-lagged autoregressive and other longitudinal modeling approaches.

Third, our models did not include potentially important mediators, such as child nutrition, health behaviors, or lifestyle habits, which could further explain the link between parenting factors, coparenting, and child metabolic health outcomes. Including these pathways in future studies would provide a more comprehensive understanding of how early family processes influence child health. Similarly, future research should adopt broader models that integrate multiple other aspects of parenting and parent-child interactions, such as parental sensitivity, disciplinary strategies, shared routines, or emotional attunement, to more fully elucidate the dynamic interplay among parental adjustment (e.g., stress and efficacy), coparenting quality, and child health. Finally, the current sample was predominantly White, educated, and middle-class, limiting the generalizability of our findings. In addition, most parents in the present sample remained cohabiting, limiting our ability to examine how coparenting processes may unfold in families with greater variability in family structure. Future studies should replicate these results in more diverse populations to enhance the broader applicability of these findings.

## Conclusion

The current study examined whether parenting stress and parental efficacy among first-time fathers and mothers during infancy are linked to children’s later metabolic health, and whether coparenting quality during toddlerhood mediates these associations. Findings revealed no direct links between parenting stress or efficacy and child metabolic outcomes. However, for mothers, coparenting quality emerged as an intervening pathway: Higher maternal stress was linked to lower-quality coparenting, whereas greater maternal efficacy was associated with more supportive coparenting. In turn, mother-reported coparenting quality was negatively associated with child blood glucose levels and BMI. Notably, when both maternal parenting stress and maternal efficacy were included in the same model, only maternal stress remained significantly associated with coparenting quality. Although several associations between paternal parenting stress, self-efficacy, and coparenting were observed, neither father parenting nor coparenting variables were significantly associated with child health outcomes. Taken together, these findings highlight the key role of coparenting in family processes, suggesting that it may be part of the pathways linking early parenting experiences, such as maternal stress, with children’s health outcomes. Although our design precludes any determination of causality, this work offers valuable insights that could inform the development of preventive interventions and family policies that support early family relationships, especially during the transition to parenthood, when parents are navigating new caregiving responsibilities. The differing findings between mothers and fathers also underscore the need for continued research that more fully captures fathers’ roles in coparenting dynamics, father–child interactions, and their contributions to child health.

## Data Availability

No datasets were generated or analysed during the current study.
